# Multicenter Validation of the CamGFR Model for Estimated Glomerular Filtration Rate

**DOI:** 10.1093/jncics/pkz068

**Published:** 2019-09-19

**Authors:** Edward H Williams, Claire M Connell, James M J Weaver, Ian Beh, Harry Potts, Cameron T Whitley, Nicholas Bird, Tamer Al-Sayed, Phillip J Monaghan, Martin Fehr, Richard Cathomas, Gianfilippo Bertelli, Amy Quinton, Paul Lewis, Jonathan Shamash, Peter Wilson, Michael Dooley, Susan Poole, Patrick B Mark, Michael A Bookman, Helena Earl, Duncan Jodrell, Simon Tavaré, Andy G Lynch, Tobias Janowitz

**Affiliations:** 1 Cancer Research UK Cambridge Institute, University of Cambridge, Cambridge, UK; 2 Cambridge University Hospital NHS Trust, Cambridge, UK; 3 Department of Oncology, University of Cambridge, UK; 4 The Christie NHS Foundation Trust, Manchester, UK; 5 The Christie Pathology Partnership, The Christie NHS Foundation Trust, Manchester, UK; 6 University of Manchester, Faculty of Medical and Human Sciences, Institute of Inflammation and Repair, Manchester, UK; 7 Western General Hospital, Edinburgh, UK; 8 School of Clinical Medicine, University of Cambridge, Cambridge, UK; 9 Clinic for Medical Oncology and Hematology, Cantonal Hospital St Gallen, Switzerland; 10 Division of Oncology/Hematology, Cantonal Hospital Graubünden, Switzerland; 11 Sussex Cancer Centre, Brighton and Sussex University Hospitals NHS Trust, Brighton, UK; 12 Velindre Cancer Centre, Cardiff, UK; 13 Institute of Life Science, Medical School, Swansea University, Swansea, UK; 14 Department of Medical Oncology, St Bartholomew's Hospital, West Smithfield, London, UK; 15 Alfred Health, Melbourne, Australia; 16 Monash University, Melbourne, Australia; 17 Institute of Cardiovascular and Medical Sciences, University of Glasgow, UK; 18 Kaiser Permanente San Francisco, San Francisco, CA; 19 NIHR Cambridge Biomedical Research Centre, Cambridge, UK; 20 Columbia University, New York, NY; 21 School of Medicine/School of Mathematics and Statistics, University of St Andrews, St Andrews, UK; 22 Cold Spring Harbor Laboratory, Cold Spring Harbor, New York, NY; 23 Northwell Health, New York, NY

## Abstract

Important oncological management decisions rely on kidney function assessed by serum creatinine–based estimated glomerular filtration rate (eGFR). However, no large-scale multicenter comparisons of methods to determine eGFR in patients with cancer are available. To compare the performance of formulas for eGFR based on routine clinical parameters and serum creatinine not calibrated with isotope dilution mass spectrometry, we studied 3620 patients with cancer and 166 without cancer who had their glomerular filtration rate (GFR) measured with an exogenous nuclear tracer at one of seven clinical centers. The mean measured GFR was 86 mL/min. Accuracy of all models was center dependent, reflecting intercenter variability of isotope dilution mass spectrometry–creatinine measurements. CamGFR was the most accurate model for eGFR (root-mean-squared error 17.3 mL/min) followed by the Chronic Kidney Disease Epidemiology Collaboration model (root-mean-squared error 18.2 mL/min).

Knowledge of kidney function measured as the glomerular filtration rate (GFR) informs clinical practice (1). GFR can be accurately measured (mGFR) using exogenous nuclear tracer clearance, but in practice, is frequently estimated (eGFR) using models based on routine clinical and biochemical data, specifically serum creatinine concentration. Creatinine is commonly measured using Jaffe or enzymatic methods, which in turn are calibrated using an isotope dilution mass spectrometry (IDMS) standard or a non–IDMS standard (2).

Recently, we derived a new model for GFR (CamGFR) using data from patients with cancer treated at the Cambridge University Hospitals NHS Foundation Trust, United Kingdom (3). CamGFR modeled GFR on a square root scale using non-IDMS–creatinine and biometric patient data and estimated GFR more accurately compared with other published models. This gain increased accuracy in GFR-based carboplatin chemotherapy dose calculations (3). Here we validate these findings for non-IDMS–creatinine-based estimation of GFR using multicenter data from patients with and without cancer.

Data were from the University Hospitals NHS Foundation Trusts in Cambridge, Southampton (4), and Manchester; Barts Health NHS Trust, London; a combined Welsh dataset (5,6); Western General Hospital, Edinburgh; and the Peter MacCallum Cancer Centre, Melbourne (7). Data on age, sex, height, weight, serum creatinine concentration, histopathologically confirmed cancer diagnosis, ethnicity, and mGFR were obtained. Either chromium-51–labeled ethylenediamine tetraacetic acid (^51^Cr-EDTA) or 99mTc-diethylenetriaminepentaacetic acid (^99m^Tc-DTPA) clearance was used to measure GFR (8,9). Serum creatinine was determined by enzymatic or Jaffe methods within 30 days of the mGFR date (Supplementary Table S1, available online). Adult patients with creatinine levels between 0.20 mg/dL and 4.5 mg/dL were included. From patients with multiple mGFR values, we included only the first value by date. Body surface area (BSA) was calculated using the DuBois and DuBois equation (10). The study was conducted at each institution according to its relevant regulatory and ethical requirements.

We compared mGFR with eGFR provided by six published models (CamGFR [3]; Martin [11]; Wright [12]; Mayo [13]; Modification of Diet in Renal Disease version 186 [14]; and Chronic Kidney Disease Epidemiology Collaboration [CKD-EPI] [15]), along with two models for creatinine clearance (Cockcroft-Gault [16] and Jelliffe [17]).

To assess model performance, statistics were determined for bias (residual median), precision (residual interquartile range [IQR]), and accuracy (root-mean-squared error [RMSE]) and clinical robustness, by calculating the proportion of patients with an absolute percentage error greater than 20% (1-P20) for eGFR; 95% confidence intervals and *P* values were approximated using bootstrap resampling (18).

Data from 3786 patients were included: A total of 3484 patients had solid cancer, 136 had hematological cancer, and 166 had a noncancer diagnosis (Table 1). Creatinine values and mGFR were obtained the same day for 27% and within 1 week for 89% of patients (Supplementary Figure S1, available online). The median mGFR was 85 mL/min (IQR = 61–109 mL/min). The median serum creatinine value was 0.95 mg/dL (IQR = 0.83–1.11 mg/dL). The median age, height, weight and BSA were 60 years, 169 cm, 74 kg, and 1.85 m^2^ respectively (Table 2). Center-specific summary statistics are provided in Supplementary Figures S2–S4 and Supplementary Tables S2–S3, available online.

**Table 1.** Characteristics of study patients: summary of categorical variables split by center

Center	Total	Solid cancer	Hematological cancer	Noncancer	Female	Race, black
Cambridge	404	227	114	63	198	6
Edinburgh	597	472	22	103	245	0
London-Barts	108	108	0	0	0	0
Manchester	1777	1777	0	0	1066	16
Melbourne	308	308	0	0	111	0
Southampton	436	436	0	0	0	0
Wales	156	156	0	0	89	0
Total	3786	3484	136	166	1709	22

**Table 2.** Characteristics of study patients: summary of continuous variables for all patients*

Variable	Mean	SD	Minimum	Q1	Median	Q3	Maximum
GFR, mL/min	86	32	9	61	85	109	209
Creatinine, mg/dL	0.99	0.28	0.43	0.83	0.95	1.11	4.45
Age, years	57	16	18	45	60	70	91
Weight, kg	76	19	33	63	74	87	200
Height, cm	169	11	137	160	169	177	204
BSA, m^2^	1.85	0.25	1.17	1.68	1.85	2.02	3.17

*GFR was measured using either ^99m^Tc-DTPA (Edinburgh and Melbourne) or ^51^Cr-EDTA (all others). ^51^Cr-EDTA = chromium-51–labeled ethylenediamine tetraacetic acid; GFR = glomerular filtration rate; BSA = body surface area (calculated using DuBois and DuBois); Q1 = 25th percentile; Q3 = 75th percentile; ^99m^Tc-DTPA = 99mTc-diethylenetriaminepentaacetic acid.

CamGFR was statistically significantly more accurate in estimating GFR than all other models, both by RMSE or 1-P20, followed by the CKD-EPI model (Figure 1, Supplementary Figure S6 and Supplementary Table S5, available online). The RMSE for the CamGFR model was 17.3 mL/min (confidence interval [CI] = 16.7 to 17.9 mL/min) and 18.2 mL/min (CI = 17.6 to 18.7 mL/min) for the CKD-EPI model (*P* = .03) and the 1-P20 results for CamGFR were 0.295 (CI = 0.280 to 0.309) and 0.318 (CI = 0.303 to 0.333) for CKD-EPI, respectively (*P* = .03). In subgroup analyses, CamGFR was the most accurate model for most patient subgroups divided by tumor type, age, BSA, serum creatinine, or sex (Supplementary Figures S7–S10, available online).

**Figure 1.**
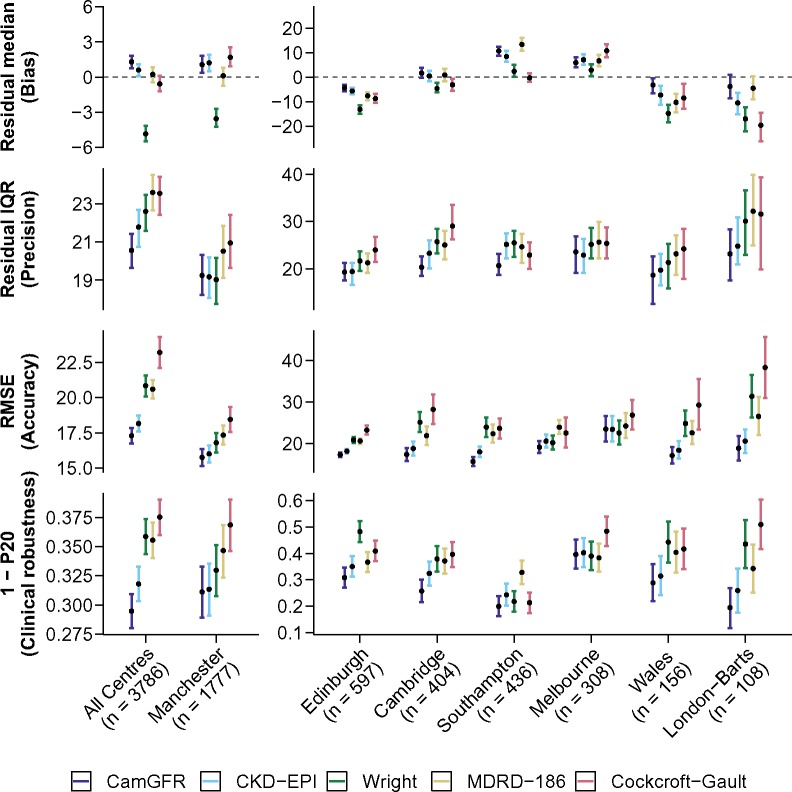
Performance analysis of commonly used and well-performing models. Results for the five best-performing models (CamGFR, CKD-EPI, Wright, MDRD-186, and Cockcroft-Gault) for the 3776 patients from the non-IDMS–creatinine validation dataset are displayed. Performance analysis of the other models is included in Supplementary Table S5 (available online). A pooled analysis of data from all centers and the individual center analyses are shown (first row). The residual (measured GFR–estimated GFR) median, which is a measure of a model’s bias, is displayed (second row). The residual interquartile range (IQR), which is a measure of a model’s precision, is displayed (third row). The RMSE, which is a measure of a model’s accuracy, is displayed. Accuracy is a combination metric of bias and precision (fourth row). The proportion of patients who have an absolute percentage error more than 20% (1-P20), which reflects clinical robustness by illustrating the proportion of patients with a clinically relevant error, is displayed. The best results are closest to zero for the residual median and the smallest value for IQR, RMSE, and 1-P20. All error bars are 95% confidence intervals calculated using bootstrap resampling with 2000 repetitions and a normal distribution approximation. CKD-EPI = Chronic Kidney Disease Epidemiology Collaboration; IDMS = isotope dilution mass spectrometry; MDRD-186 = Modification of Diet in Renal Disease version 186; RMSE = root-mean-squared error.

Finally, CamGFR had the lowest RMSE both for male and female patients and in six out of seven centers. Model performance was not consistent between centers (Supplementary Table S5, available online, Figure 1), probably reflecting differences in non-IDMS–creatinine values (Supplementary Figure S3, available online).

We did not adjust the CamGFR model to include race as a potential variable for two reasons: the small number of black patients (n = 22) and the absence of a statistically significant difference in BSA, mGFR, or serum creatinine when we compared 10 random data draws matched for age and sex between nonblack and black patients (Supplementary Figure S5 and Supplementary Table S4, available online). Other studies have documented systematic differences for the relationship between eGFR and creatinine for black patients (14,15), and our study is probably underpowered to detect this. The use of non-IDMS–creatinine data in this study represents a further limitation (19). Differences between non-IDMS and IDMS creatinine exist (2), and future work should expand the CamGFR model to IDMS–creatinine data use. Of note, the CKD-EPI model was developed for use with IDMS–creatinine measurements specifically, but it still outperformed other models that have been developed with non-IDMS data.

The data were mostly from chemotherapy treatment–naive patients with cancer, and the longitudinal effect of treatment on eGFR requires further study. Probably attributable to the near-normal renal function of the majority of the patients in our study, we find that the underlying diagnosis of the patients does not affect the suitability of the models. CamGFR, developed on data from patients with cancer, performs best in noncancer patients, and CKD-EPI, developed on data from patients without cancer, performs well for data from patients with cancer.

This work is based on data from seven centers, and it confirms that of the available models, the CamGFR model estimates GFR most accurately, but the CKD-EPI model performs nearly as well overall and across the spectrum of relevant subgroups. The greatest gain in accuracy by these newer models over the older models, such as Cockcroft-Gault and Wright, was observed in younger patients and patients with lower creatinine values, probably reflecting the differences in model-development populations. However, even considering the different patient populations in different centers, it is likely that errors in estimating GFR can be reduced by standardizing the methods used to measure serum creatinine at different laboratories and using appropriate models. Given the linear relationship between GFR and carboplatin dose via the Calvert equation (20), improved estimates of GFR using CamGFR will translate into carboplatin prescriptions that are more accurate.

## Funding

This work was supported by Cancer Research UK (EHW, TJ: C42738/A24868); National Institute of Health Research Cambridge Biomedical Research Centre (HE); National Institute of Health Research UK Academic Clinical Fellowship (CMC); and National Institutes of Health USA Cancer Center support grant (TJ: 5P30CA045508-31).

## Notes

Affiliations of authors: Cancer Research UK Cambridge Institute, University of Cambridge, Cambridge, UK (EHW, CMC, DJ, ST, AGL, TJ); Cambridge University Hospital NHS Trust, Cambridge, UK (CMC, NB, HE, DJ); Department of Oncology, University of Cambridge, UK (CMC, HE); The Christie NHS Foundation Trust, Manchester, UK (JMJW, TA-S); The Christie Pathology Partnership, The Christie NHS Foundation Trust, Manchester, UK (PJM); University of Manchester, Faculty of Medical and Human Sciences, Institute of Inflammation and Repair, Manchester, UK (PJM); Western General Hospital, Edinburgh, UK (IB); School of Clinical Medicine, University of Cambridge, Cambridge, UK (HP, CTW); Clinic for Medical Oncology and Hematology, Cantonal Hospital St Gallen, Switzerland (MF); Division of Oncology/Hematology, Cantonal Hospital Graubünden, Switzerland (RC); Sussex Cancer Centre, Brighton and Sussex University Hospitals NHS Trust, Brighton, UK (GB); Velindre Cancer Centre, Cardiff, UK (AQ); Institute of Life Science, Medical School, Swansea University, Swansea, UK (PL); Department of Medical Oncology, St Bartholomew's Hospital, West Smithfield, London, UK (JS, PW); Alfred Health, Melbourne, Australia (MD, SP); Monash University, Melbourne, Australia (MD, SP); Institute of Cardiovascular and Medical Sciences, University of Glasgow, UK (PBM); Kaiser Permanente San Francisco, San Francisco, CA (MAB); NIHR Cambridge Biomedical Research Centre, Cambridge, UK (HE); Columbia University, New York, NY (ST); School of Medicine/School of Mathematics and Statistics, University of St Andrews, St Andrews, UK (AGL); Cold Spring Harbor Laboratory, Cold Spring Harbor, New York, NY (TJ); Northwell Health, New York, NY (TJ).

We thank all the patients. We also thank the reviewers, the University of Cambridge, Cancer Research UK, and Hutchison Whampoa Limited.

CamGFR is available online at https://sites.google.com/site/janowitzwilliamsgfr/.

## Supplementary Material

pkz068_Supplementary_Data
