# Supplementary material for: Multicenter Validation of the CamGFR Model for Estimated Glomerular Filtration Rate
Source: JNCI Cancer Spectr. 2019 Sep 19;3(4):pkz068. doi: 10.1093/jncics/pkz068 (PMC6846361; doi:10.1093/jncics/pkz068)
Supplement: pkz068_Supplementary_Data [file pkz068_supplementary_data.pdf]

Supplement for: Multicentre validation of the CamGFR model for estimated glomerular filtration rate

Abstract

This document is the supplemental file for the paper "Multicentre validation of the CamGFR model for estimated glomerular filtration rate" and provides additional methods, results, figures and tables. The .Rmd file format contains the R code used for this manuscript. Table S5 contains all results, but for space reasons only Table S5a is included in this document. The entire Table S5 and all R files are available on request and deposited at github.com/EdwardHWilliams/CamGFRValidationNonIDMS.

Data description and filtering

Raw data that were received from the seven different centres, which are identified by their location (Cambridge, Edinburgh, London-Barts, Manchester, Melbourne, Southampton and Wales). Data were individually processed and cleaned prior to being used for analyses. This step involved exploring the data for manual transcription errors such as the height and weight variable being swapped and removing unrealistic values for some variables. During this process all data were converted to the same units to enable data pooling and analyses across the different centres and subgroup categories.

Next, patients with data outside the inclusion criteria were removed. The inclusion criteria were:

- Serum creatinine value between 18 μmol/L (0.2 mg/dL) and 400 μmol/L (4.5 mg/dL)
- Age of 18 years or older
- Creatinine and GFR measurements performed within 30 days of each other
- If a patient had multiple GFR measurements, only the first one would be included

The creatinine cut-off values were chosen because the lower value is the typical detection threshold on the measurement assay and the upper value is 3-4 times the upper limit of normal in most centres, thus corresponding to a value at which most clinicians would consider kidney function severely impaired. The 30 days was selected to minimise errors caused by changing creatinine. We confirmed that a 30 day window before and after mGFR was reasonable for creatinine measurements by comparing accuracy in seven different windows relating to the difference in measurement dates. We detected no change in accuracy (Figure S11).

All creatinine data were either measured using a non-IDMS traceable measurement or the measurement procedure was not known, but likely to be non-IDMS traceable due to the dates of measurements. Table S1 summaries the information on the creatinine measurement for each centre.

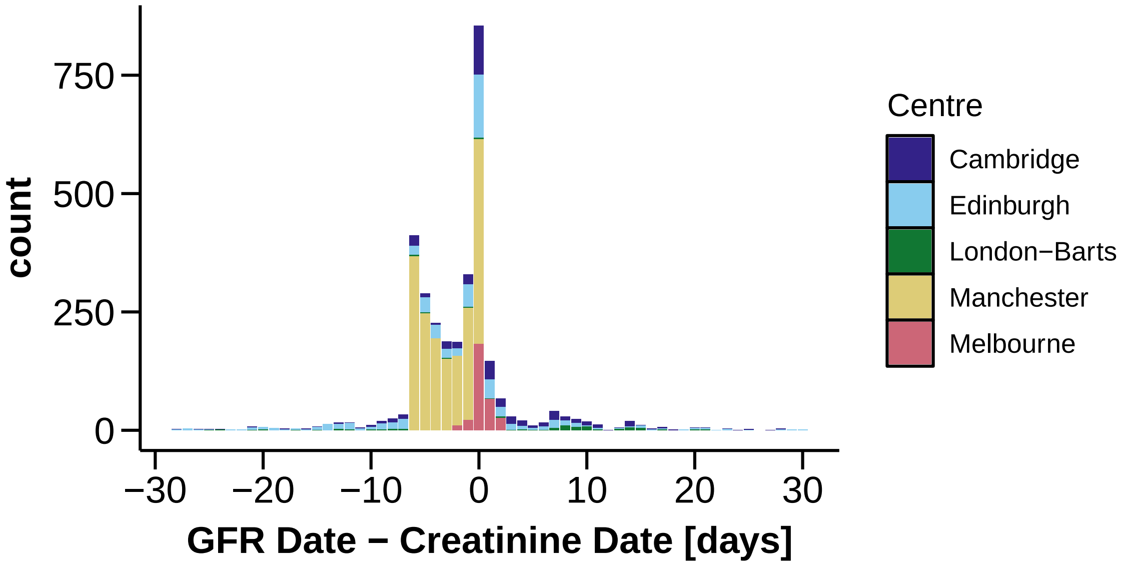

Figure S1: Barplot of the time difference between GFR and serum creatinine measurements for each patient. A negative difference indicates that the serum creatinine was measured after the GFR. Some centres only reported time ranges for the creatinine, i.e. that it was measured within 30 days of GFR, and not specific dates. Data from these centres were not included for this barplot.

Table S1: Creatinine methods used at each centre

Centre	Date range	Creatinine measurement method
Cambridge	2013	Dimension RxL compensated Jaffe, not IDMS-traceable
Edinburgh	2011 - 2017	An Abbott Architect assay using the Jaffe method; not IDMS-traceable
London-Barts	2010 - 2017	Unknown method; IDMS usage not known
Manchester	2012 - 2016	Jaffe method using O'Leary reagent; not IDMS-traceable
Melbourne	2000 - 2005	Jaffe method; not IDMS-traceable
Southampton	1999 - 2012	Jaffe method; given the date range, most, if not all, not IDMS-traceable
Wales	2005 - 2011, not all dates known	Different centres used either Jaffe or enzymatic methods; IDMS usage not known

Figure S1 shows the difference in time between the GFR and creatinine measurements days. Most patients had their creatinine measured on, or in, the week after the nuclear medicine GFR, with this being the case for all patients from Manchester.

Table 1 in the main manuscript provides the number of patients in different categorical groups and the summary statistics for the continuous variables (GFR, age, serum creatinine, body surface area (BSA), height and weight) for all patients. Here we provide the summary statistics for patients split into different centres (Table S2).

Table S2: Summary characteristics from each centre

	Mean	SD	Minimim	Q1	Median	Q3	Maximum
**Cambridge**							
GFR [ml/min]	87	29	9	66	87	107	177
Creatinine [µmol/L]	87	28	40	70	83	97	258
Age [years]	54	16	18	43	55	66	86
Weight [kg]	77	19	39	64	75	88	184
Height [cm]	170	10	146	162	169	178	200
BSA [m²]	1.87	0.24	1.34	1.7	1.86	2.02	2.87
**Edinburgh**							
GFR [ml/min]	83.7	35	9.1	54.4	83	110.2	206
Creatinine [µmol/L]	90	32	45	70	81	102	393
Age [years]	56	16	18	43	58	68	87
Weight [kg]	79	19	38	66	78	90	197
Height [cm]	171	10	144	164	171	179	202
BSA [m²]	1.91	0.24	1.27	1.73	1.91	2.06	3.17
**London-Barts**							
GFR [ml/min]	110	20	46	98	111	122	166
Creatinine [µmol/L]	81	14	52	73	82	88	141
Age [years]	39.2	9.2	22.4	32.7	38.7	45.1	70.8
Weight [kg]	85	17	57	73	82	90	150
Height [cm]	177.9	7.8	160	172.8	177.8	182	200
BSA [m²]	2	0.2	1.6	1.9	2	2.1	2.6
**Manchester**							
GFR [ml/min]	77	29	14	56	74	94	194
Creatinine [µmol/L]	88	22	46	74	83	96	235
Age [years]	62	14	18	54	65	72	91
Weight [kg]	72	19	33	59	70	83	164
Height [cm]	164.6	9.8	137	157	164	171	200
BSA [m²]	1.78	0.24	1.17	1.6	1.76	1.93	2.79
**Melbourne**							
GFR [ml/min]	85	31	17	64	83	104	205
Creatinine [µmol/L]	96	30	60	80	90	110	290
Age [years]	63	13	18	56	65	72	87
Weight [kg]	73	15	37	62	72	80	145
Height [cm]	169.4	9.3	148	163	170	176.6	194
BSA [m²]	1.83	0.21	1.31	1.69	1.83	1.96	2.62
**Southampton**							
GFR [ml/min]	119	21	51	104	118	132	209
Creatinine [µmol/L]	89	12	54	80	89	97	136
Age [years]	39.9	9.5	19	33	39	46	75
Weight [kg]	86	16	54	76	85	95	200
Height [cm]	179	7	156	174	179	183	204

Table S3: Number of patients with given diagnoses in each centre

Diagnosis	Manchester	Edinburgh	Cambridge	Southampton	Melbourne	Wales	London-Barts	Total
**Cancer**								
GCT	35	118	56	436	0	67	108	820
Unknown	437	8	45	0	308	0	0	798
Gynae	251	54	74	0	0	89	0	468
Bladder/TCC	88	138	25	0	0	0	0	251
Thoracic	219	13	3	0	0	0	0	235
Gastro-oesophageal	203	19	5	0	0	0	0	227
Head and Neck	192	3	6	0	0	0	0	201
Endometrial	104	21	0	0	0	0	0	125
Uterus/Cervix	83	15	1	0	0	0	0	99
Sarcoma	31	34	10	0	0	0	0	75
Breast	29	6	2	0	0	0	0	37
HPB	26	6	0	0	0	0	0	32
Colorectal	17	10	0	0	0	0	0	27
CUP	26	1	0	0	0	0	0	27
RCC	12	6	0	0	0	0	0	18
Prostate	5	8	0	0	0	0	0	13
Neuroendocrine	1	11	0	0	0	0	0	12
Anal	6	1	0	0	0	0	0	7
CNS	6	0	0	0	0	0	0	6
Skin	5	0	0	0	0	0	0	5
Melanoma	1	0	0	0	0	0	0	1
**Haematological**								
Haematological	0	22	114	0	0	0	0	136
**Non-cancer**								
Unknown	0	4	0	0	0	0	0	4
Kidney donor	0	94	35	0	0	0	0	129
Transplant	0	0	28	0	0	0	0	28
Unknown	0	5	0	0	0	0	0	5

Table S2: Summary characteristics from each centre *(continued)*

	Mean	SD	Minimim	Q1	Median	Q3	Maximum
BSA [m²]	2.05	0.19	1.62	1.92	2.03	2.16	3.09
**Wales**							
GFR [ml/min]	89	31	23	65	90	111	169
Creatinine [μmol/L]	74	19	38	61	74	84	164
Age [years]	57	18	22	42	58	72	90
Weight [kg]	77	18	45	63	76	88	155
Height [cm]	168	11	145	160	166	177	192
BSA [m²]	1.86	0.24	1.41	1.67	1.83	2.04	2.57

To facilitate data comparison graphically, Figure S2 displays the data from Table S2 in several boxplots. Patients from Southampton and London-Barts have a higher GFR, height and weight and a lower age than patients from other centres. This is attributable to the fact that the data from these centres were from patients with seminoma only and

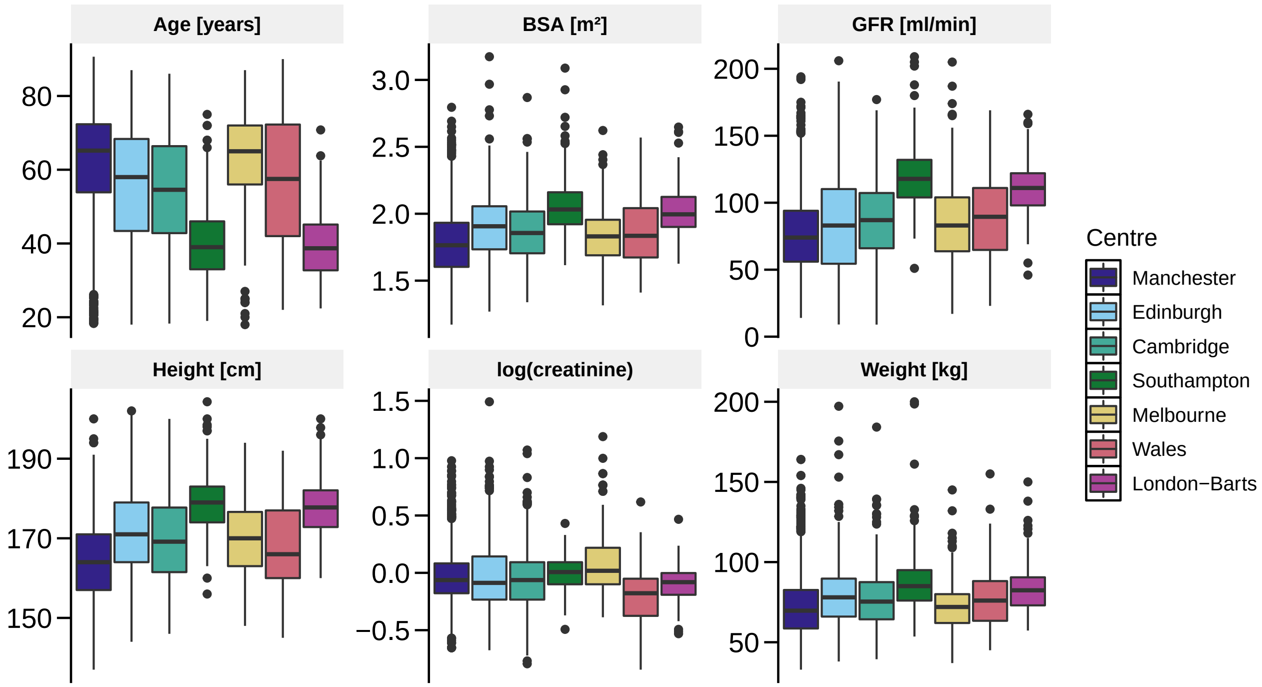

Figure S2: Boxplot of the continuous variables split by centre. Creatinine has been log transformed.

hence were typically from young men. The panel of log(creatinine) illustrates the inter-centre variability.

Figure S3 show boxplots for log(creatinine) split by centre for all patients and patients with selected diagnoses respectively. As patients with similar diagnoses should be approximately matched for the other variables, the plots facilitate further comparison of serum creatinine differences between centres.

Figure S4 takes the same format as Figure S2 but displays subgroups of patients based on diagnosis. Subgroups with more than 50 patients were included.

Information on ethnicity was available for patients from Cambridge and Manchester, who comprised the majority of patients. From a total of 2,017 patients with known ethnicity, 1,910 were White (either White British, White Irish, White other), 70 were Asian (Asian Indian, Asian Pakistani, Asian Bangladeshi, Asian other, Chinese, Other Chinese, Mixed White and Asian), 22 were Black (Black African, Black Caribbean, Black other, Mixed White and Black Caribbean, Mixed White and Black African) and 15 were of other ethnicity (other ethnic group, other mixed). Figure S5 shows boxplots for age, BSA, mGFR and log(creatinine) for patients from the ethnic subgroups. As white patients were the largest subgroup, t-tests were performed to look for differences between this subgroup and each other subgroup. There were no significant differences in the serum creatinine levels for patients from different ethnicities. However, there were significant differences in the other variables and therefore the subgroups were not matched. Given the correlations between these variables, it is difficult to interpret the creatinine comparisons precisely. To determine whether the average creatinine values differ between white and black patients when the other variables are similar, we sampled 22 white patients from the population that matched the set of black patients with regards to sex and age ten times. We then performed t-tests for these ten samples for log(creatinine), measured GFR and BSA. No significant differences were found (Table S4).

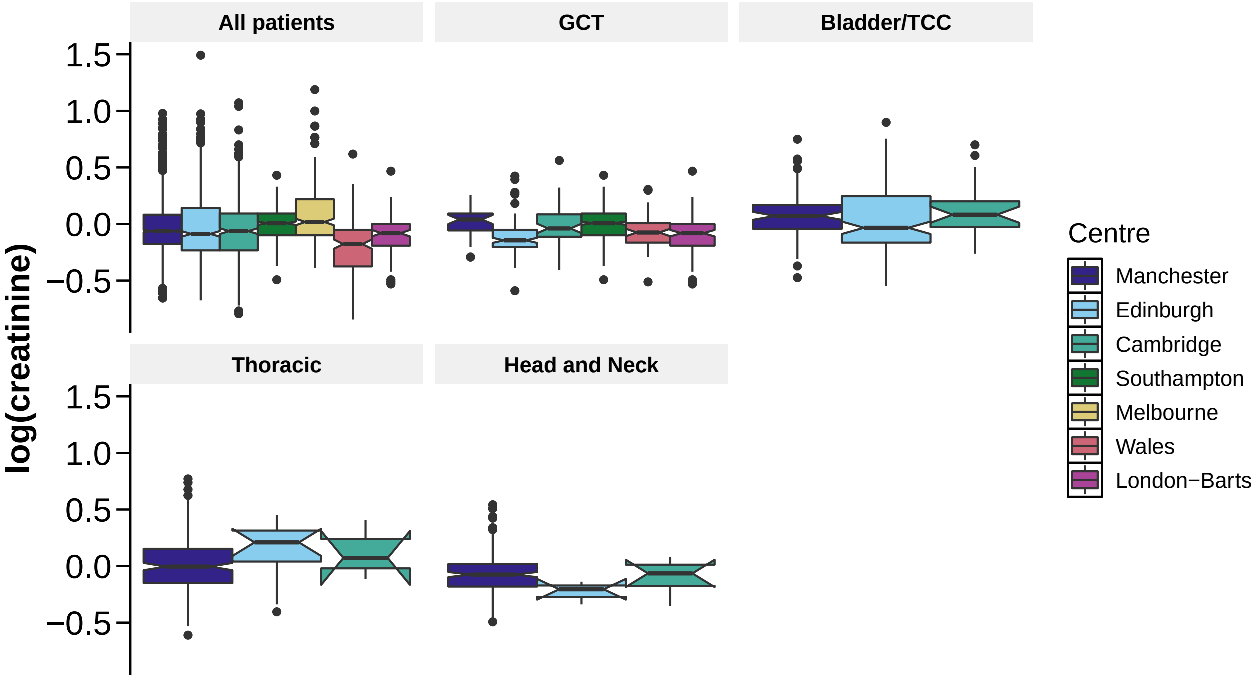

Figure S3: Boxplots of the serum creatinine of patients from different centres. Boxplots are shown for all patients combined and for patients with Germ Cell Tumours (GCT), Bladder or Transitional cell cancer (TCC), Thoracic cancer, Gastro-oesophageal cancer and Head and Neck cancer. The notches in the boxplots are 1.58IQR$/\sqrt{n}$ (where n is the number of patients used for the given boxplot and IQR is the inter quartile range); they provide both an approximate 95% confidence interval for the median and an indication of the number of patients used for the given boxplot.

Table S4: t-test p-values for comparing the mean age, log(creatinine), GFR and BSA between 22 patients who were ethnically black and 22 random patients who were ethnically white and matched by mean age (mean age within 1 year) and the same gender distribution (12 females and 10 males).

Age [years]	log(creatinine)	GFR [ml/min]	BSA [m²]
0.98	0.39	0.72	0.88
0.92	0.29	0.61	0.16
0.93	0.01	0.98	0.06
0.89	0.89	0.91	0.93
0.91	0.51	0.92	0.57
0.90	0.53	0.59	0.83
0.85	0.67	0.79	0.88
0.86	0.44	0.74	0.78
0.97	0.31	0.65	0.68
0.94	0.38	0.10	0.19

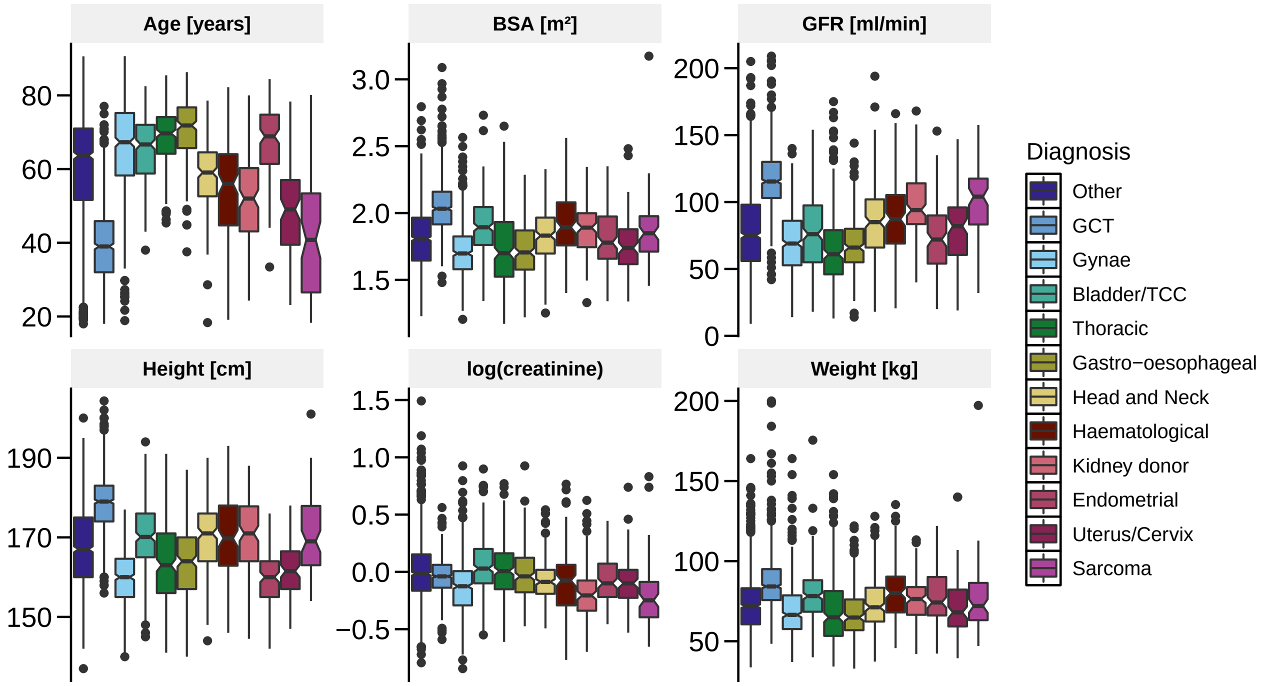

Figure S4: Boxplot of the continuous variables split by diagnosis. Subgroups with more than 50 patients are displayed. Subgroups with fewer than 50 patients have been combined with patients with an unknown diagnosis into the group Other. Boxplots are arranged by decreasing patient numbers from left to right. Serum creatinine has been log transformed.

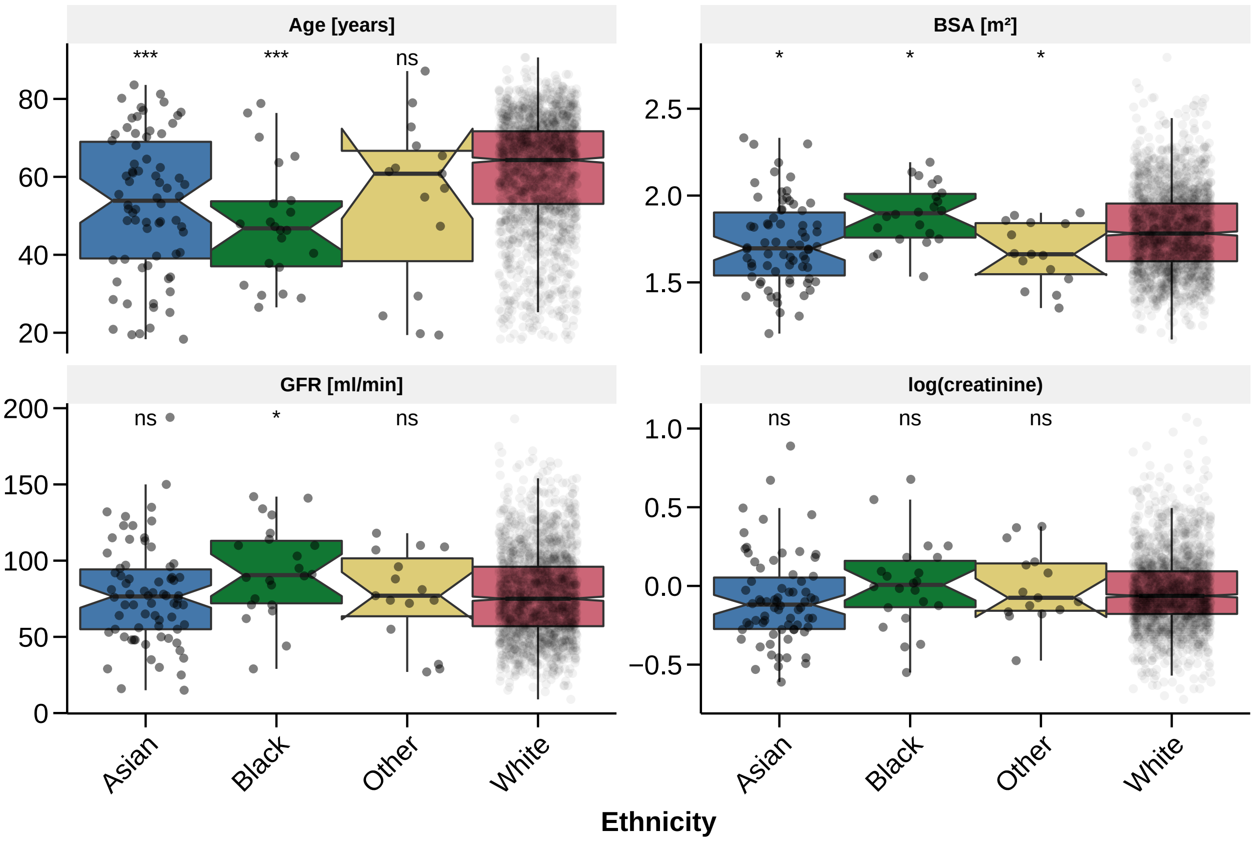

Figure S5: Boxplots of age, BSA, GFR and log(creatinine) and Age where patients are split by ethnicity. The '*' or 'ns' at the top of the boxplot represents the p-value from a t-test between the largest subgroup of white patients and each other ethnic subgroups, where 'ns' represents a p-value greater than 0.05 and '*', '**' and '***' represent p-values less than 0.05, 0.01 and 0.001 respectively.

Comparison of models

We compared the CamGFR[1] model with the the following published models: CKD-EPI[2], MDRD (186 version)[3], Wright[4], Cockcroft-Gault[5], Jelliffe[6], Mayo[7] and Martin[8]. If these equations estimated BSA-normalised GFR (CKD-EPI, MDRD, Jelliffe and Mayo), the estimated value was subsequently unnormalised by multiplying the estimated value by $1.73/$BSA, where BSA is the patient's body surface area estimated using the DuBois-DuBois equation:

$$\text{BSA} = 0.20247 \times \text{Height}^{0.725} \times \text{Weight}^{0.425}$$

where height is measured in metres and weight is measured in kg. Some of these models (Cockroft-Gault and Jelliffe) were developed to estimate creatinine clearance and not GFR, where creatinine clearance is itself a biased estimator of GFR. These models are included as they are still used to estimate GFR by some centres, clinical trial groups, and in other settings.

The equations were compared using three metrics: the root-mean-squared-error (RMSE), median residual value, and the residual interquartile range (IQR). These three metrics are estimators for a model's accuracy, bias and precision respectively. A 95% confidence interval was calculated for each of these metrics using the bootstrap resampling procedure. Specifically, 2,000 resamples with replacement (where the sample size was the same as the number of data points) were taken from the data used to calculate the metric. The metric was then calculated for each of these 2,000 samples and using the normal approximation a confidence interval was constructed[9]. The same seed was used for different metrics.

The main results of this analysis are reported in Figure 1 (results for patients split by centre) in the main manuscript along with Figures S7 (results for patients split by diagnosis), S8 (results for patients split by age) and S9 (results for patients split by BSA). Additionally, all numerical results are reported in Table S5 (an additional file). Table S5 gives the results for all equations above whereas the Figures only compare the performance of CamGFR, CKD-EPI, MDRD, Wright and Cockroft-Gault.

Additionally, we compared the performance of the CamGFR and CKD-EPI models with the aid of a Bland-Altman plot (or MA plot) and residual vs fitted plots (Figure S6). The Bland-Altman plot is used to contrast accuracy, whereas the fitted vs residual plot is used to to assess the performance of a model. The feature which is most noticeable from Figure S6 is that for low eGFR (approximately less than 50 ml/min), CamGFR estimates larger values than CKD-EPI. The inverse is true for high eGFR, culminating in a sight overall bias for eGFR to be higher in the CKD-EPI than CamGFR. The variability between the two models increases as eGFR increases. This can be partly explained by the fact that CamGFR and CKD-EPI model GFR on a square root and log scale respectively. The fitted vs residual plots are similar for the two models.

We analysed performance when patients were split into groups by their age or BSA. Figures S8, S9, and S10 show the performance of each model for equally sized subgroups of age, BSA, and creatinine respectively. The accuracy of all models tends to increase with increasing age and tends to increase with increasing BSA. These observations can be largely explained by the fact that GFR is modelled on the square root scale in the CamGFR model and on the log scale for the CKD-EPI or MDRD models. Patients who are young or have a large BSA also tend to have a higher GFR and patients with a higher GFR will have a less accurate estimated GFR due to modelling on a square-root or log scale. As discussed in the main manuscript, CamGFR is not the most accurate for the subgroups split by serum creatinine. For the lower two groups CamGFR, is the most accurate model for all subgroups split by serum creatinine. For the two groups with lower creatinine, CamGFR is the most accurate model. In particular, for the group of patients with the lowest serum creatinine values all other models are biased to overestimate GFR.

P-values for the differences between a statistic for a pair of equations were calculated using a bootstrap procedure described by Efron[10]. In this procedure we have two samples $\mathbf{z}$ and $\mathbf{y}$ of lengths n and m respectively, from possibly

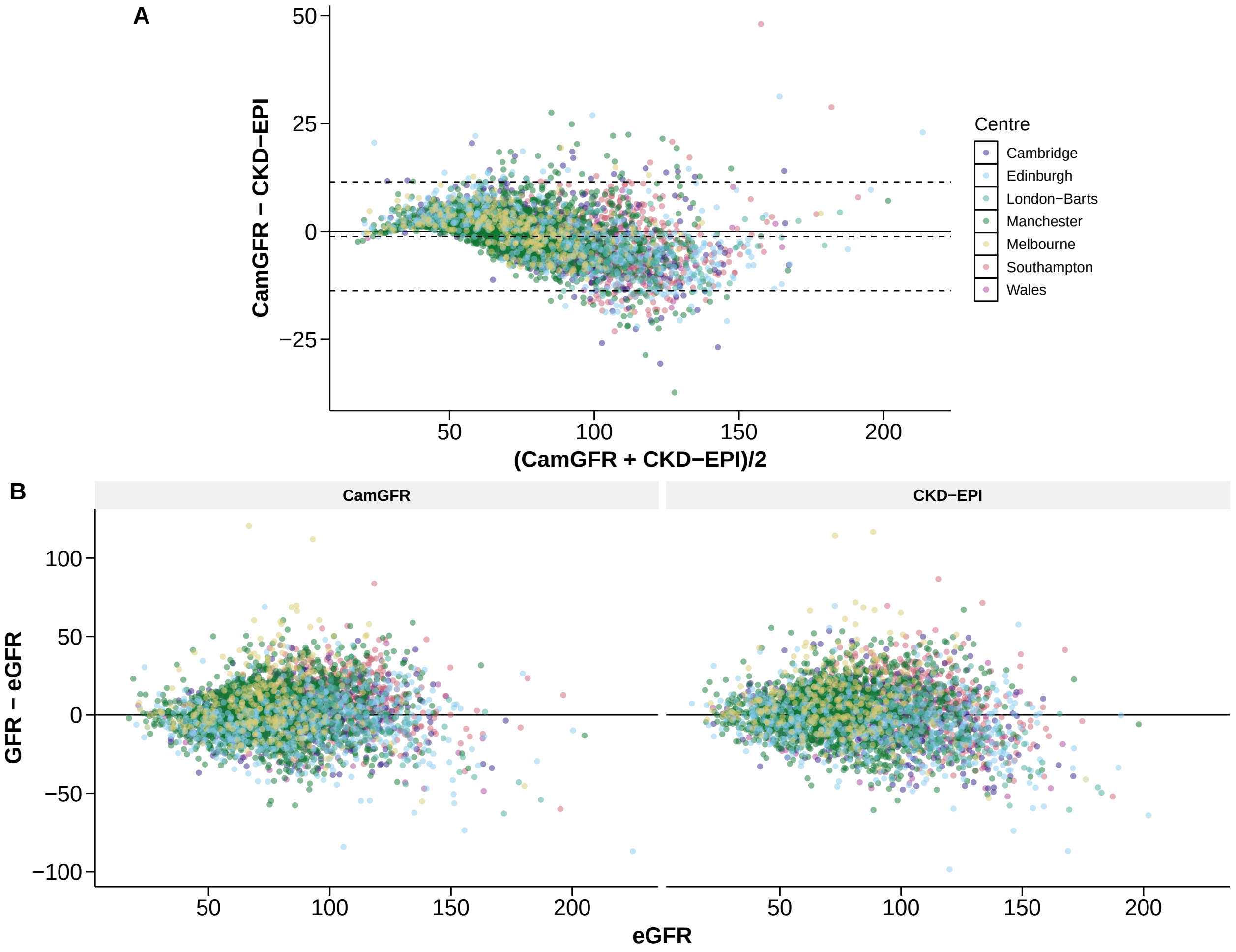

Figure S6: Comparison of GFR estimated by CamGFR and CKD-EPI, the two best performing model. A) Bland-Altman plot of GFR estimated using the CamGFR and CKD-EPI models. B) Residual (GFR - eGFR) vs fitted value (eGFR) for both the CamGFR and CKD-EPI models.

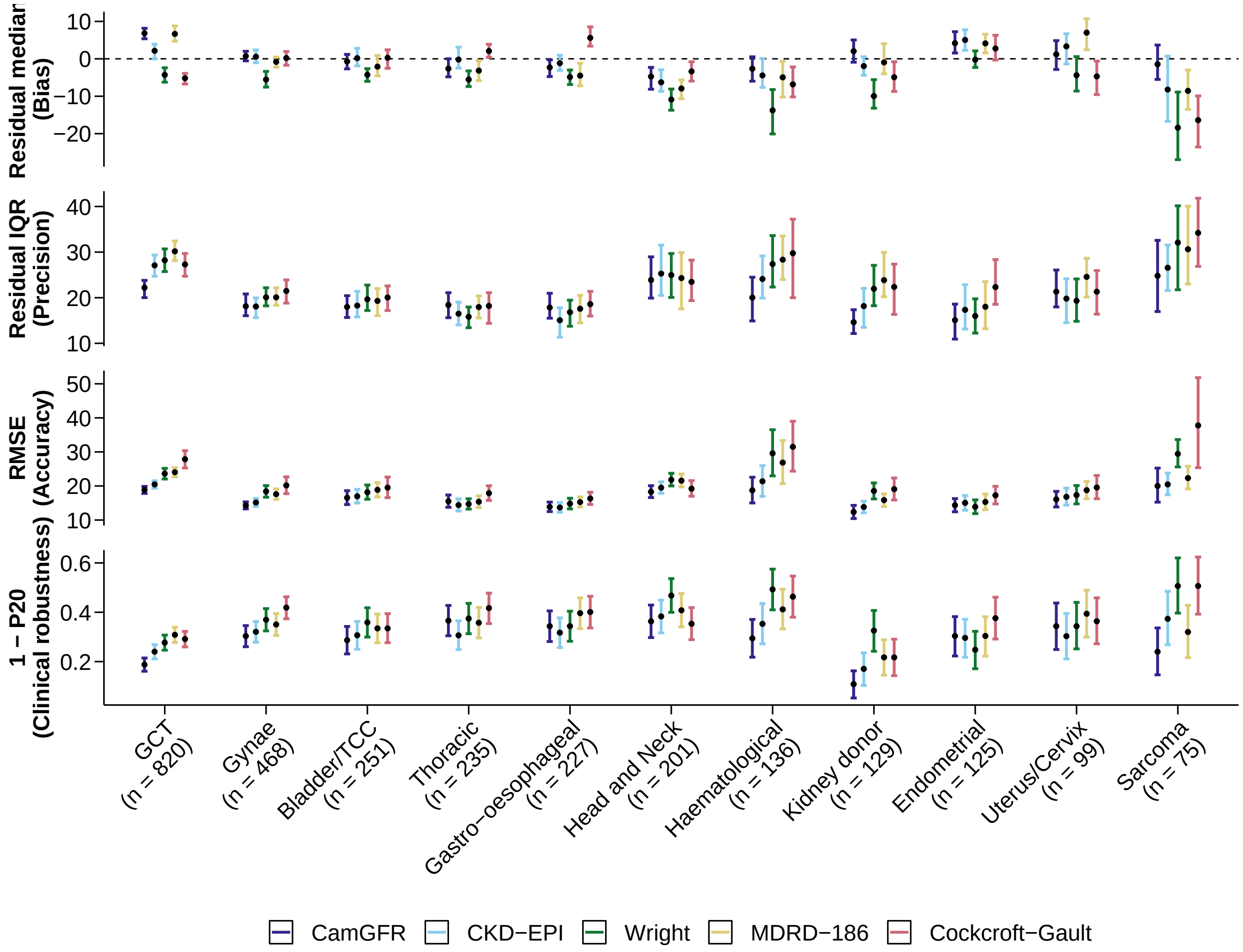

Figure S7: Performance of GFR models for patients from different diagnostic subgroups. The results for the CamGFR model and four other commonly used models (CKD-EPI, Wright, MDRD-186 and Cockcroft-Gault) are displayed. Results for those diagnostic subgroups that had more than 50 patients are shown. First row: the residual (measured GFR - estimated GFR) median, which is a measure of a model's bias, is displayed. Second row: the residual interquartile range (IQR), which is a measure of a model's precision, is displayed. Third row: the root-mean-squared error (RMSE), which is a measure of a model's accuracy, is displayed. Accuracy is a combination metric of bias and precision. Fourth row: The proportion of patients who have an absolute percentage error more than 20% (1-P20), which reflects clinical robustness by illustrating the proportion of patients with a clinically relevant error, is displayed. The best result would be closest to zero for the residual median, and the smallest value for IQR, RMSE, and 1-P20. All error bars are 95% confidence intervals calculated using bootstrap resampling with 2,000 repetitions and a normal distribution approximation.

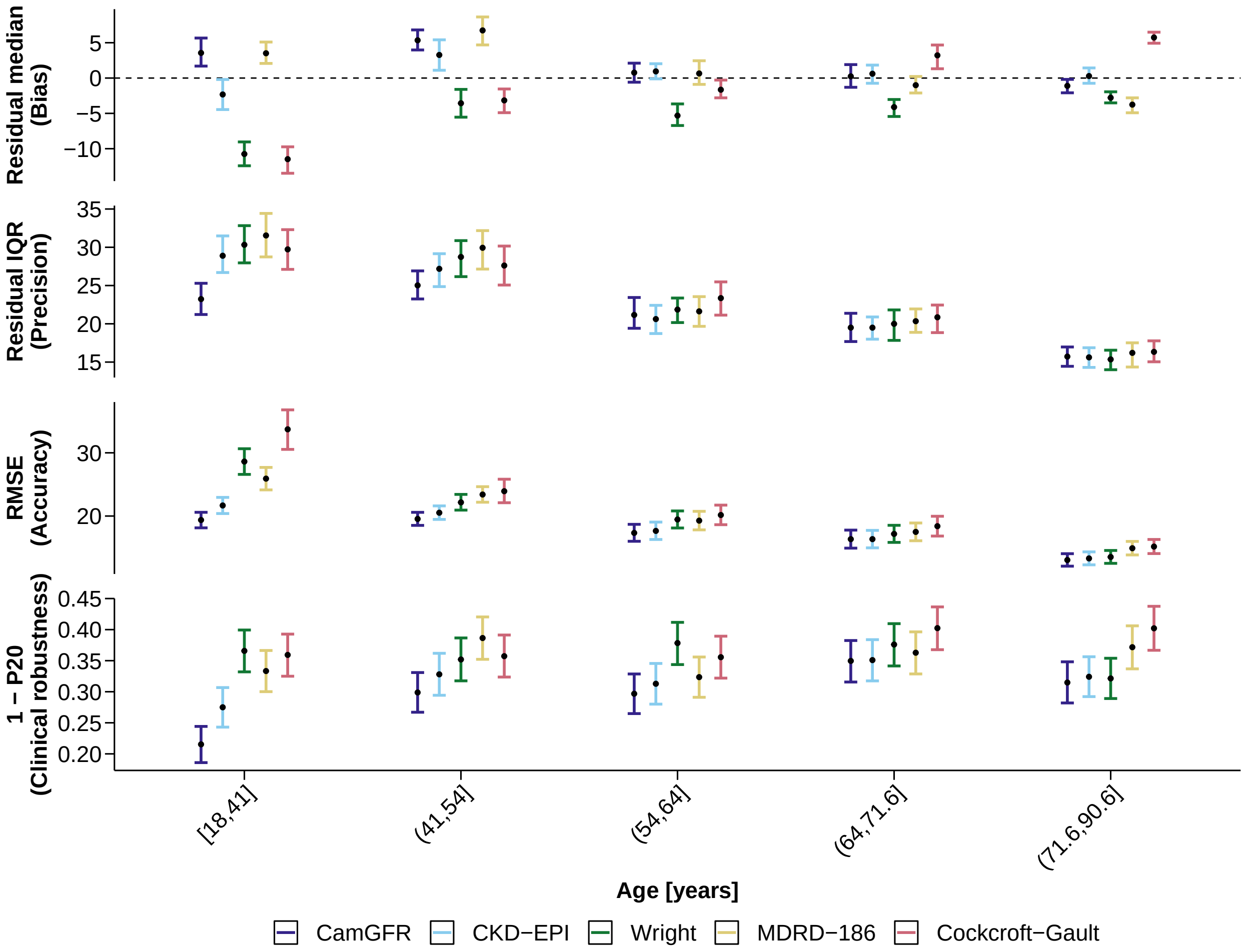

Figure S8: Comparison of model performance in different age groups. The results for five published models are shown. Patients were split into 10 equally sized groups by their age. (*a*,*b*] denotes the interval *a* to *b* which is exclusive of the lower and inclusive of the higher age.

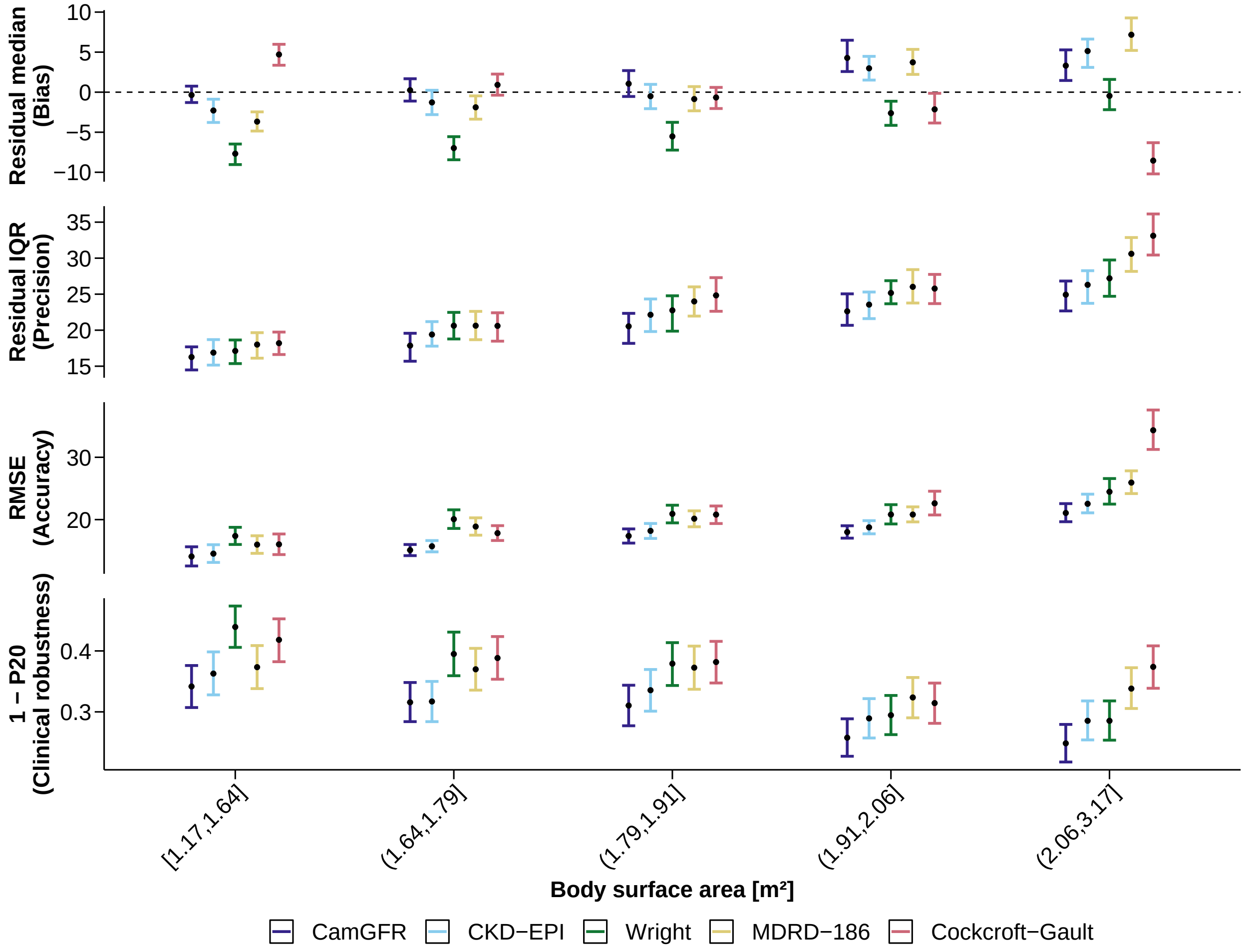

Figure S9: Comparison of model performance in different BSA groups. The results for five published models are shown. Patients were split into 5 equally sized groups by their BSA. (*a*,*b*] denotes the interval *a* to *b* which is exclusive of the lower and inclusive of the higher BSA.

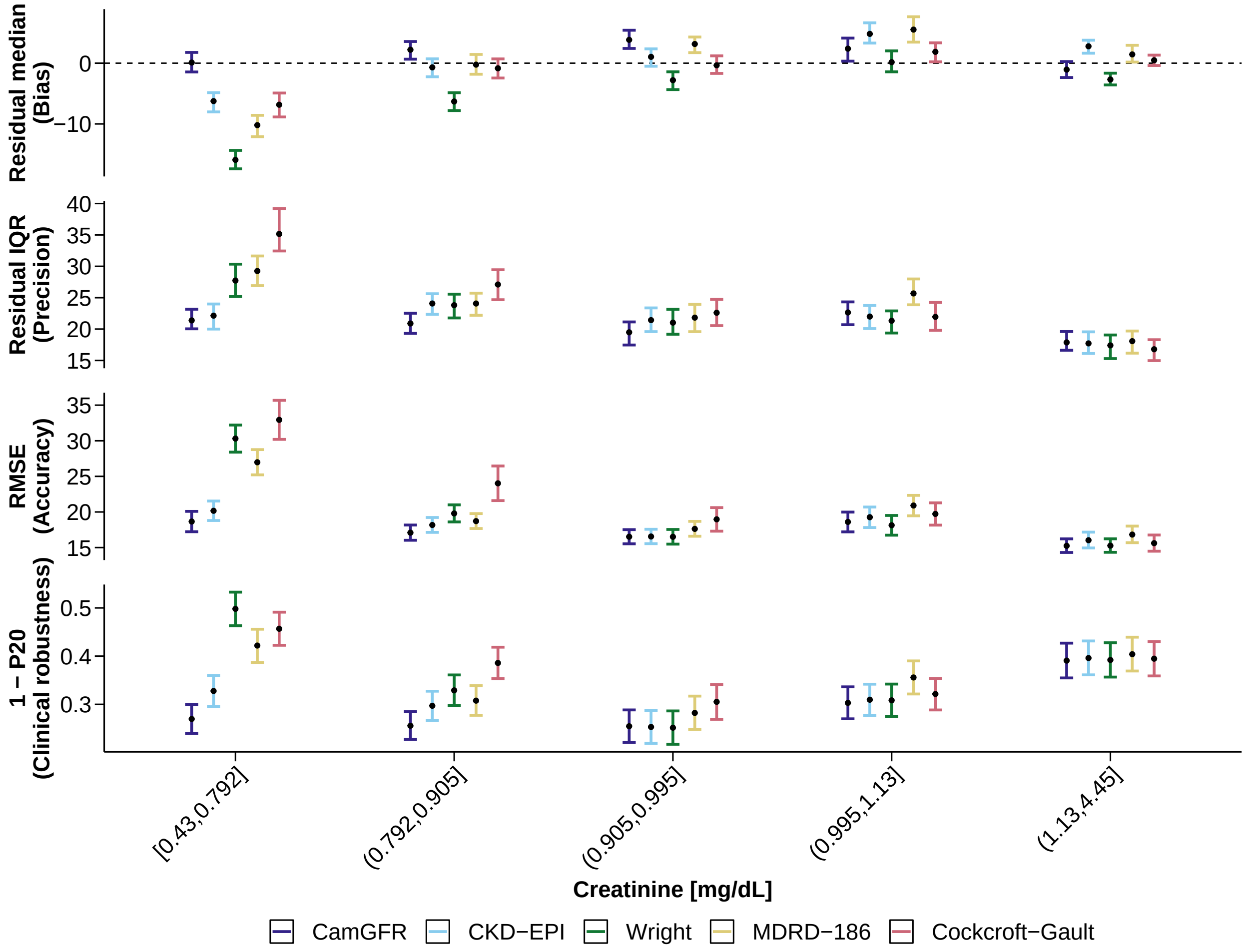

Figure S10: Comparison of model performance in different serum creatinine groups. The results for five published models are shown. Patients were split into 5 equally sized groups by their serum creatinine. (*a*,*b*] denotes the interval *a* to *b* which is exclusive of the lower and inclusive of the higher BSA.

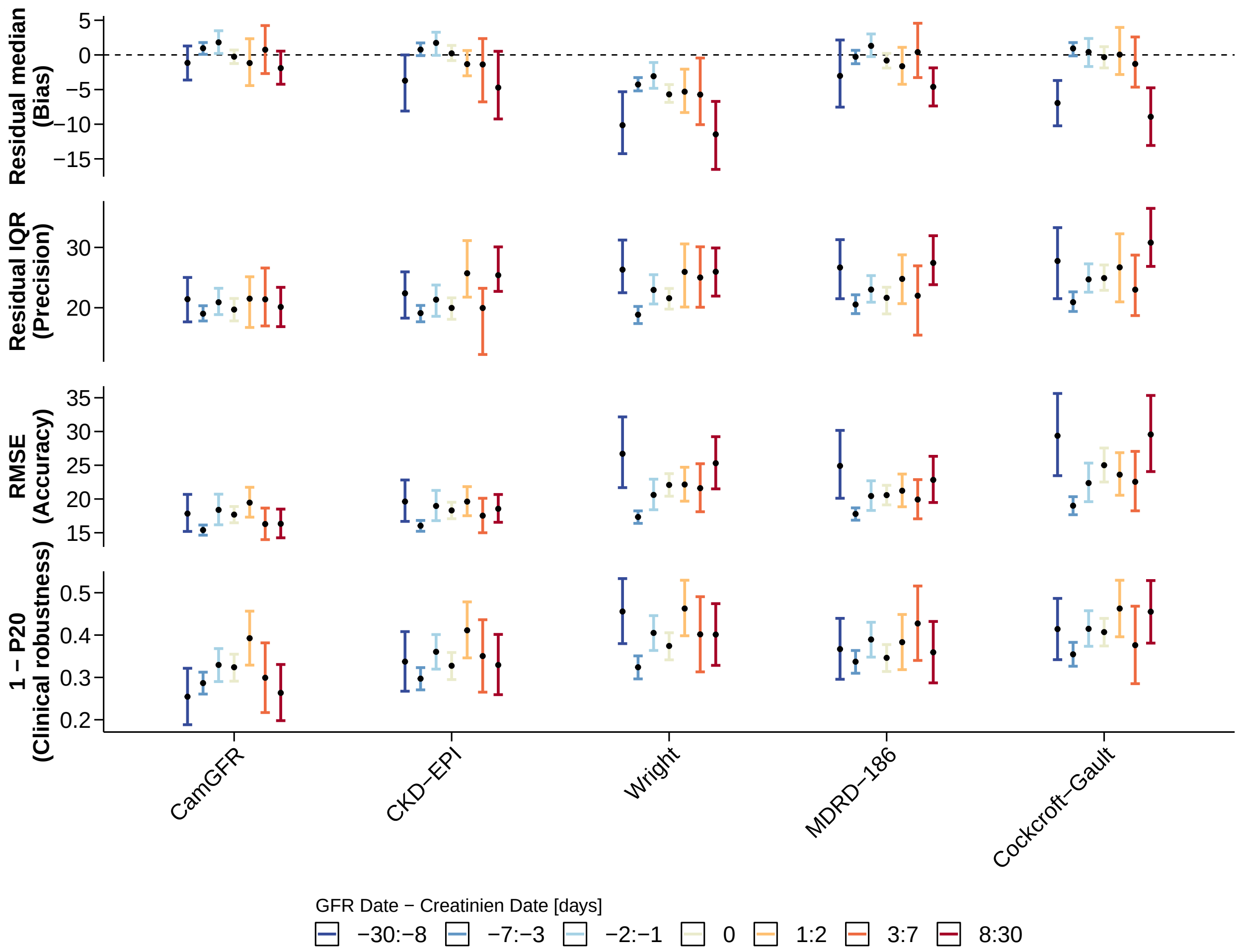

Figure S11: Comparison of model performance patients split by the difference in time between the GFR and creatinine measurement dates. The results for five published models are shown. A negative date difference corresponds to creatinine measured after GFR.

different probability distributions F and G. In our case F and G were the probability distributions of the residuals for the two equations of interest. We tested the null hypothesis $H_0 : F = G$, against the alternative hypothesis $H_0 : F \neq G$. Let $x = [z, y]$, and chose a test statistic $t(\mathbf{x})$, which had the form $t(\mathrm{x}) = f(\mathrm{z}) - f(\mathrm{y})$, where the function $f(\mathrm{w})$ was either RMSE, median or IQR. Using the following procedure an approximate p-value was calculated by:

- For $r = 1, ..., R$ let x_r^* be a sample with replacement of size $n + m$ from the pooled vector $\mathrm{x} = [\mathrm{z}, \mathrm{y}]$. Let z_r^* be the first n observations of x_r^* and y_r^* the remaining m observations.
- Let
$$t_r^* = t(\mathrm{x}_r^*) = f(\mathrm{z}_r^*) - f(\mathrm{y}_r^*), \quad r = 1, 2, ..., R$$
- Calculate the approximate p-value for the hypothesis test as:
$$\widehat{p} = \frac{1 + \sum_{r=1}^{R} \Bbbk_{|t_r^*| \geq |t_{obs}|}}{R + 1}$$
where $t_{obs} = t(\mathrm{x}) = f(\mathrm{z}) - f(\mathrm{y})$ is the observed value of the statistic and $\Bbbk_A$ is the indicator function which returns 1 if A is true and 0 otherwise. The absolute values of t are considered so as to perform a two-sided test.

It should be noted that that the hypothesis test is strictly testing the null hypothesis $H_0 : F = G$ and not the null hypothesis $H_0 : f(Z) = f(G)$. When the approximate p-values are calculated with the statistic 1-P20 (the proportion of patients with an absolute percentage error more than 20%), z and y would be the vectors of percentage differences between the fitted and measured dose values. Approximate p-values (with $R = 10,000$) are given in Table S5 (an additional file).

Table S5: Results and p-value tables for all equations analysed

a) Results for all data

equation	n	median fitted value	RMSE	median	IQR	1-P20
CamGFR	3786	82.36	17.3 (16.73, 17.85)	1.3 (0.74, 1.84)	20.56 (19.65, 21.42)	0.295 (0.28, 0.309)
CKD-EPI	3786	83.39	18.16 (17.58, 18.72)	0.6 (0.08, 1.11)	21.78 (20.75, 22.68)	0.318 (0.303, 0.333)
MDRD-186	3786	83.08	20.61 (19.93, 21.26)	0.23 (-0.47, 0.85)	23.59 (22.66, 24.52)	0.356 (0.34, 0.371)
Wright	3786	88.16	20.85 (20.1, 21.59)	-4.86 (-5.5, -4.17)	22.6 (21.59, 23.47)	0.359 (0.343, 0.374)
Jelliffe	3786	73.13	22.2 (21.54, 22.86)	9.37 (8.72, 9.98)	23.84 (22.88, 24.83)	0.44 (0.424, 0.456)
Cockcroft-Gault	3786	82.78	23.23 (22.11, 24.32)	-0.58 (-1.23, 0.11)	23.55 (22.43, 24.41)	0.375 (0.36, 0.391)
Martin	3786	90.93	25.23 (24.07, 26.38)	-7.89 (-8.61, -7.18)	24.06 (22.84, 25.01)	0.412 (0.396, 0.427)
Mayo	3786	99.59	25.89 (25.27, 26.49)	-16.77 (-17.51, -16.07)	22.98 (22, 23.88)	0.539 (0.523, 0.554)

Published models

This section gives the published models used in this manuscript. In the following equations:

- Age is the patient's Age in years
- BSA is the patient's body surface area in m^2, if is estimated for the patients weight and weight using the DuBois DuBois equation [@Janowitz2017a]
- Scr is the patient's blood serum creatinine in mg/dL
- Wt is the patient's weight is kg

CamGFR[1]

$$GFR = \Big(1.81395 + 0.01914 \times Age + 4.73278 \times BSA - 0.02970 \times Age \times BSA - 3.71619 \times \text{log}\,(Scr) + 1.06284 \times \text{log}\,(Scr)^2 - 0.91420 \times \text{log}\,(Scr)^3 + (0.02020 + 0.01247 \times Age)\,[\text{if male}]\Big)^2$$

CKD-EPI[2]

$$GFR = \begin{cases} 141 \times \text{min}\left(\frac{Scr}{0.7}, 1\right)^{-0.329} \times \text{max}\left(\frac{Scr}{0.7}, 1\right)^{-1.209} \times Age^{0.993} \times 1.018 & \text{if Sex = F} \\ 141 \times \text{min}\left(\frac{Scr}{0.9}, 1\right)^{-0.411} \times \text{max}\left(\frac{Scr}{0.9}, 1\right)^{-1.209} \times Age^{0.993} & \text{if Sex = M} \end{cases}$$

Cockcroft Gault[5]

$$GFR = \frac{(140 - Age) \times Wt \times (1 - 0.15[\text{if female}])}{72 \times Scr}$$

MDRD-186[11]

$$GFR = 186 \times Scr^{-1.154} \times Age^{-0.203} \times 0.742[\text{if female}]$$

Jelliffe[6]

$$GFR = \frac{(98 - 0.8(Age - 20)) \times (1 - 0.1[\text{if female}]) \times \frac{BSA}{1.73}}{Scr}$$

Wright[4]

$$GFR = \frac{(6580 - 38.8 \times Age) \times (1 - 0.168[\text{if female}]) \times BSA}{88.42 * Scr}$$

Mayo Quadratic[7]

$$GFR = \exp\left(1.911 + 5.249\frac{1}{Scr} - 2.114\frac{1}{Scr^2} - 0.00686Age - 0.205[\text{if female}]\right)$$

if $Scr < 0.8$mg/dL then use 0.8 for Scr

Martin[8]

$$GFR = \frac{163 \times \mathrm{Wt}\ \times (1 - 0.00496 \times \mathrm{Age}) \times (1 - 0.252[\text{if female}])}{88.42 \times \mathrm{Scr}}$$

References

1. Janowitz T, Williams EH, Marshall A, et al. New model for estimating glomerular filtration rate in patients with cancer. *Journal of Clinical Oncology : official journal of the American Society of Clinical Oncology*. 2017;35(24):2798-2805. doi:10.1200/JCO.2017.72.7578

2. Levey AS, Stevens LA, Frcp C, et al. A new equation to estimate glomerular filtration rate. *Annals of Internal Medicine*. 2009;150(9):604-612. doi:10.7326/0003-4819-150-9-200905050-00006

3. Levey AS, Bosch JP, Lewis JB, Greene T, Rogers N, Roth D. A more accurate method to estimate glomerular filtration rate from serum creatinine: a new prediction equation. *Annals of Internal Medicine*. 1999;130(6):461-470. doi:10.7326/0003-4819-130-6-199903160-00002

4. Wright JG, Boddy AV, Highley M, Fenwick J, McGill A, Calvert AH. Estimation of glomerular filtration rate in cancer patients. *British Journal of Cancer*. 2001;84(4):452-459. doi:10.1054/bjoc.2000.1643

5. Cockcroft DW, Gault MH. Prediction of creatinine clearance from serum creatinine. *Nephron*. 1976;16(1):31-41. http://www.ncbi.nlm.nih.gov/pubmed/1244564.

6. Jelliffe RW. Creatinine clearance: bedside estimate. *Annals of Internal Medicine*. 1973;79(4):604-605. doi:10.7326/0003-4819-79-4-604

7. Rule AD, Larson TS, Bergstralh EJ, Slezak JM, Jacobsen SJ, Cosio FG. Using serum creatinine to estimate glomerular filtration rate: accuracy in good health and in chronic kidney disease. *Annals of internal medicine*. 2004;141(12):929-937. doi:10.7326/0003-4819-141-12-200412210-00009

8. Martin L, Chatelut E, Boneu A, et al. Improvement of the Cockcroft-Gault equation for predicting glomerular filtration in cancer patients. *Bulletin du Cancer*. 1998;85(7):631-636. http://www.ncbi.nlm.nih.gov/pubmed/9752271.

9. Davison AC, Hinkley DV. *Bootstrap Methods and Their Application*. Cambridge University Press; 1997.

10. Efron B, Tibshirani RJ. *An Introduction to the Bootstrap*. New York: Chapman & Hall; 1994.

11. Levey A, Coresh J, Greene T, et al. Using Standardized Serum Creatinine Values in the Modification of Diet in Renal Disease Study Equation for Estimating Glomerular. *Annals of Internal Medicine*. 2006;145:247-254. doi:10.7326/0003-4819-145-4-200608150-00004
